# Machine Learning-Based Phenomapping in Patients with Heart Failure and Secondary Prevention Implantable Cardioverter-Defibrillator Implantation: A Proof-of-Concept Study

**DOI:** 10.31083/j.rcm2402037

**Published:** 2023-02-02

**Authors:** Yu Deng, Sijing Cheng, Hao Huang, Xi Liu, Yu Yu, Min Gu, Chi Cai, Xuhua Chen, Hongxia Niu, Wei Hua

**Affiliations:** ^1^Cardiac Arrhythmia Center, State Key Laboratory of Cardiovascular Disease, Fuwai Hospital, National Center for Cardiovascular Diseases, Chinese Academy of Medical Sciences & Peking Union Medical College, 100037 Beijing, China

**Keywords:** heart failure, implantable cardioverter-defibrillator, secondary prevention, machine learning-based phenomapping, the first appropriate shock, all-cause death

## Abstract

**Background:**

Previous studies have failed to implement risk stratification in patients with heart failure (HF) who are eligible for secondary implantable cardioverter-defibrillator (ICD) implantation. We aimed to evaluate whether machine learning-based phenomapping using routinely available clinical data can identify subgroups that differ in characteristics and prognoses.

**Methods:**

A total of 389 patients with chronic HF implanted with an ICD were included, and forty-four baseline variables were collected. Phenomapping was performed using hierarchical k-means clustering based on factor analysis of mixed data (FAMD). The utility of phenomapping was validated by comparing the baseline features and outcomes of the first appropriate shock and all-cause death among the phenogroups.

**Results:**

During a median follow-up of 2.7 years for device interrogation and 5.1 years for survival status, 142 (36.5%) first appropriate shocks and 113 (29.0%) all-cause deaths occurred. The first 12 principal components extracted using the FAMD, explaining 60.5% of the total variability, were left for phenomapping. Three mutually exclusive phenogroups were identified. Phenogroup 1 comprised the oldest patients with ischemic cardiomyopathy; had the highest proportion of diabetes mellitus, hypertension, and hyperlipidemia; and had the most favorable cardiac structure and function among the phenogroups. Phenogroup 2 included the youngest patients, mostly those with non-ischemic cardiomyopathy, who had intermediate heart dimensions and function, and the fewest comorbidities. Phenogroup 3 had the worst HF progression. Kaplan–Meier curves revealed significant differences in the first appropriate shock (*p* = 0.002) and all-cause death (*p <* 0.001) across the phenogroups. After adjusting for medications in Cox regression, phenogroups 2 and 3 displayed a graded increase in appropriate shock risk (hazard ratio [HR] 1.54, 95% confidence interval [CI] 1.03–2.28, *p* = 0.033; HR 2.21, 95% CI 1.42–3.43, *p <* 0.001, respectively; *p* for trend <0.001) compared to phenogroup 1. Regarding mortality risk, phenogroup 3 was associated with an increased risk (HR 2.25, 95% CI 1.45–3.49, *p <* 0.001). In contrast, phenogroup 2 had a risk (*p* = 0.124) comparable with phenogroup 1.

**Conclusions:**

Machine-learning-based phenomapping can identify distinct phenotype subgroups in patients with clinically heterogeneous HF with secondary prophylactic ICD therapy. This novel strategy may aid personalized medicine for these patients.

## 1. Introduction

Evidence-based clinical guidelines recommend implantable cardioverter-defibrillator (ICD) for primary and secondary prevention of ventricular arrhythmias (VA) and sudden cardiac death (SCD) [1, 2]. In fact, most primary prevention ICD recipients derive no benefit in the subsequent follow-up [3, 4, 5]. Therefore, previous studies have focused on identifying risk factors, constructing risk assessment tools, and finding new parameters and modalities to facilitate risk stratification in these patients [4, 6, 7, 8]. In contrast, the benefit of ICD therapy in secondary prevention is more certain due to the established substrate for VA [9]. Consequently, little attention has been paid to secondary prophylactic ICD implantations. Risk stratification in these patients is imperative to understand the heterogeneity of disease development, progression, and prognosis [10].

Despite efforts being made, previous studies could not predict VA recurrence in patients undergoing secondary prevention [11, 12]. The individual risk of VA recurrence is high, irrespective of other factors [13]. Thus, in this circumstance, instead of identifying underlying risk factors, segregating patients into different subgroups based on their similarities, called phenomapping, might provide a more feasible solution [14]. In theory, patients within a phenogroup share similar baseline features and long-term prognoses, whereas patients within different phenogroups differ in these aspects. Through phenomapping, personalized treatment and management, including programming settings, medication optimization, and VA ablation, can be refined.

This study aimed to first classify patients into different phenogroups using unsupervised machine learning clustering based on principal component analysis and then validate the clinical utility of phenogroups by comparing the baseline characteristics and outcomes (including all-cause death and the first appropriate shock) among the established phenogroups.

## 2. Materials and Methods

### 2.1 Study Population

We retrospectively included all patients with heart failure (HF) who underwent secondary prophylactic single- or dual-chamber ICD implantation at our center between January 2010 and December 2020 (n = 756). The exclusion criteria were as follows: patients with cardiac channelopathies, hypertrophic cardiomyopathy, and congenital heart disease (n = 132); patients with heart failure with preserved ejection fraction (n = 172); and patients without visit after ICD implantation (n = 63). The study was performed in accordance with the Declaration of Helsinki and approved by the Fuwai Hospital. In addition, written informed consent was obtained from all the patients.

### 2.2 Outcome Assessment

All-cause of death and first appropriate ICD shock on a VA event were primary outcomes. Patient survival status was obtained from medical records, death certificates, or phone contact with their family members. Additionally, ICD discharge information was extracted using device interrogation. The patients were required to have planned interrogation at least yearly and unplanned interrogation after sensible device therapy. The ICD programming settings were at the discretion of the treating physicians. No standard protocols were requested; nonetheless, shock therapies were generally set to be delivered after ATP could not terminate the VA events. Patients were censored at the time of their successful VA ablation, and patient survival was further ascertained after their last device interrogations.

### 2.3 Data Collection and Processing

Forty-four baseline variables, including demographics, physical examination, cardiovascular risk factors, laboratory tests, electrocardiogram, echocardiographic parameters, comorbidities, and medications, were collected. All the variables had a missing rate of <3.9% (**Supplementary Table 1**). Missing values were imputed using random forests. Data standardization was applied to eliminate different scales of variables, and the original form was used for interpretation.

### 2.4 Phenotypic Clustering

To determine the natural intrinsic connection within ICD recipients, all baseline characteristics, excluding the medications (thirty-four variables in total), were used for phenomapping. Hierarchical k-means clustering was chosen for phenotypic clustering [15, 16], which includes the two most widely used unsupervised machine learning algorithms: hierarchical clustering and k-means clustering. Hierarchical clustering was first computed to determine the optimal number of clusters and their respective centroids. Subsequently, k-means clustering was performed using these centroids as the initial cluster centers. Both steps combined to make the clustering results more reliable and robust. Therefore, this is also called k-means-consolidated hierarchical clustering. It has been successfully applied in clustering patients with dilated cardiomyopathy [17].

Specifically, in this study, as it is not directly applicable to mixed data types (continuous and categorical), the factor analysis of mixed data (FAMD) was a preliminary step to clustering [15, 16]. To obliterate redundant information contained in the raw variables, only principal components extracted using the FAMD with eigenvalues >1 were retained. Based on the selected principal components, hierarchical clustering was performed using the Euclidean distance and Ward’s criterion. The optimal number of clusters (1–10) was determined by the Elbow method, using the total within the sum of squares as metrics. Finally, k-means consolidation was performed, leading to the final phenomapping.

The representative variables in each cluster were analyzed using the v test based on the hypergeometric distribution. Briefly, a positive v-test statistic indicates the overrepresentation of a variable, whereas a negative statistic indicates the underrepresentation of a variable. The baseline characteristics and clinical outcomes of the phenogroups were compared to validate the clinical utility of phenomapping.

### 2.5 Statistics

Continuous data were expressed as mean ± standard deviation or median with 25th and 75th percentiles, as appropriate; categorical data were presented as numbers and percentages. Baseline characteristics between phenogroups were compared using ANOVA or Kruskal–Wallis test for continuous variables and χ^2^ or Fisher test for categorical variables. Cumulative incidences were estimated using the Kaplan–Meier method and compared using the log-rank test. Unadjusted and adjusted relationships between clusters and outcomes were assessed using Cox proportional hazard regression and described using hazard ratios (HR) and 95% confidence intervals (95% CI). Medications were included in the adjusted models. The backward stepwise selection was applied using the Akaike information criterion to obtain the most parsimonious model. Analyses were conducted using R (version 4.1.2, R Foundation for Statistical Computing, Vienna, Austria), mainly through the “missForest”, “FactoMineR”, and “factoextra” packages. Statistical significance was set at *p <* 0.05.

## 3. Results

### 3.1 Baseline Characteristics

A total of 389 patients with HF with reduced or mildly reduced ejection fraction were identified. Table 1 summarizes the baseline characteristics of the study population during the ICD implantation. The mean age was 60.5 ± 12.5 years. The patients were predominantly male (83.5%), and approximately half had ischemic cardiomyopathy (52.4%). The mean left ventricular ejection fraction (LVEF) was 35.5 ± 8.4%, and 46.3% of patients had New York Heart Association (NYHA) Class III or IV.

**Table 1.** **Baseline clinical characteristics in study patients stratified by phenogroups**.

Characteristics		All patients (n = 389)	Phenogroup 1 (n = 163)	Phenogroup 2 (n = 148)	Phenogroup 3 (n = 78)	*p* for overall
Demographics						
	Age (years)	60.5 ± 12.5	65.2 ± 10.5	54.3 ± 12.4	62.6 ± 11.7	<0.001
	Male sex	325 (83.5%)	144 (88.3%)	112 (75.7%)	69 (88.5%)	0.005
	Body mass index (kg/$m^{2}$)	24.8 ± 3.6	25.2 ± 3.2	24.9 ± 3.8	23.9 ± 3.9	0.039
	Ischemic cardiomyopathy etiology	204 (52.4%)	155 (95.1%)	20 (13.5%)	29 (37.2%)	<0.001
	Family history of sudden death	9 (2.3%)	3 (1.8%)	6 (4.1%)	0 (0.0%)	0.141
Clinical characteristics						
	Smoking	200 (51.4%)	105 (64.4%)	62 (41.9%)	33 (42.3%)	<0.001
	Dual-chamber ICD	134 (34.4%)	73 (44.8%)	43 (29.1%)	18 (23.1%)	0.001
	Systolic BP (mmHg)	118.5 ± 16.8	124.0 ± 17.9	114.8 ± 12.4	114.0 ± 18.5	<0.001
	Diastolic BP (mmHg)	73.6 ± 10.6	74.9 ± 10.3	73.6 ± 9.1	70.9 ± 12.9	0.019
	NYHA class III/IV	180 (46.3%)	51 (31.3%)	64 (43.2%)	65 (83.3%)	<0.001
Echocardiogram						
	LVEDD (mm)	64.6 ± 8.8	62.0 ± 7.6	65.5 ± 8.1	68.3 ± 10.8	<0.001
	LVEF (%)	35.5 ± 8.4	38.3 ± 7.5	34.2 ± 8.2	32.1 ± 8.8	<0.001
	LAD (mm)	44.1 ± 7.1	42.0 ± 5.5	43.3 ± 6.1	50.1 ± 8.5	<0.001
	RVD (mm)	22.9 ± 4.3	21.9 ± 2.9	22.3 ± 3.7	26.1 ± 5.9	<0.001
	IVS (mm)	9.3 ± 1.8	9.0 ± 1.8	9.3 ± 1.8	9.6 ± 1.8	0.054
	Tricuspid valve regurgitation	26 (6.7%)	1 (0.6%)	5 (3.4%)	20 (25.6%)	<0.001
	Mitral valve regurgitation	86 (22.1%)	21 (12.9%)	22 (14.9%)	43 (55.1%)	<0.001
Electrocardiogram findings						
	Heart rate (beats per minute)	70.0 ± 13.1	68.6 ± 12.3	69.5 ± 12.6	73.8 ± 15.1	0.013
	CLBBB	27 (6.9%)	2 (1.2%)	20 (13.5%)	5 (6.4%)	<0.001
	CRBBB	27 (6.9%)	14 (8.6%)	7 (4.7%)	6 (7.7%)	0.392
	Frequent PVCs	166 (42.7%)	52 (31.9%)	81 (54.7%)	33 (42.3%)	<0.001
	Pacing indication	18 (4.6%)	3 (1.8%)	7 (4.7%)	8 (10.3%)	0.016
Comorbidities						
	Myocardial infarction	176 (45.2%)	151 (92.6%)	7 (4.7%)	18 (23.1%)	<0.001
	Atrial fibrillation	121 (31.1%)	30 (18.4%)	35 (23.6%)	56 (71.8%)	<0.001
	Hypertension	164 (42.2%)	88 (54.0%)	36 (24.3%)	40 (51.3%)	<0.001
	Diabetes	82 (21.1%)	48 (29.4%)	16 (10.8%)	18 (23.1%)	<0.001
	Hyperlipidemia	193 (49.6%)	115 (70.6%)	43 (29.1%)	35 (44.9%)	<0.001
	Stroke	31 (8.0%)	12 (7.4%)	7 (4.7%)	12 (15.4%)	0.018
	Hyperuricemia	45 (11.6%)	17 (10.4%)	7 (4.7%)	21 (26.9%)	<0.001
Laboratory tests						
	NT-proBNP (pg/mL)	1036.0 (534.2–2065.1)	805.0 (329.0–1787.0)	919.0 (542.5–1562.8)	3404.9 (1857.5–5766.4)	<0.001
	Hemoglobin (g/L)	141.1 ± 18.4	136.8 ± 19.0	146.9 ± 15.9	138.9 ± 19.0	<0.001
	Creatinine (μmol/L)	93.6 (81.0–111.0)	96.5 (83.0–113.8)	85.2 (75.8–95.0)	109.7 (96.6–135.2)	<0.001
	BUN (mmol/L)	7.2 (5.7–9.3)	7.2 (5.7–9.0)	6.6 (5.4–8.0)	9.5 (7.3–13.3)	<0.001
	hs-CRP (mg/L)	2.4 (0.9–5.6)	2.3 (0.8–4.7)	1.6 (0.8–4.2)	5.2 (2.0–11.1)	<0.001
Medications						
	ACEI/ARB/ARNI	303 (77.9%)	126 (77.3%)	122 (82.4%)	55 (70.5%)	0.118
	Amiodarone	276 (71.0%)	130 (79.8%)	94 (63.5%)	52 (66.7%)	0.005
	Beta-blockers	339 (87.1%)	152 (93.3%)	126 (85.1%)	61 (78.2%)	0.003
	Calcium channel blockers	36 (9.3%)	15 (9.2%)	15 (10.1%)	6 (7.7%)	0.834
	Diuretics	304 (78.1%)	120 (73.6%)	114 (77.0%)	70 (89.7%)	0.017
	MRA	285 (73.3%)	116 (71.2%)	116 (78.4%)	53 (67.9%)	0.177
	Digitalis	94 (24.2%)	22 (13.5%)	36 (24.3%)	36 (46.2%)	<0.001
	Statin	207 (53.2%)	133 (81.6%)	44 (29.7%)	30 (38.5%)	<0.001
	Antiplatelet	152 (39.1%)	110 (67.5%)	26 (17.6%)	16 (20.5%)	<0.001
	Anticoagulants	75 (19.3%)	21 (12.9%)	24 (16.2%)	30 (38.5%)	<0.001

Values are presented as the mean ± standard deviation, median (interquartile range), or frequency (%). ACEI/ARB/ARNI, angiotensin-converting enzyme inhibitor/angiotensin receptor blocker/angiotensin receptor-neprilysin inhibitor; BP, blood pressure; BUN, blood urea nitrogen; CLBBB, complete left bundle branch block; CRBBB, complete right bundle branch block; hs-CRP, high-sensitivity C-reactive protein; ICD, implantable cardioverter-defibrillator; IVS, interventricular septum thickness; LAD, left atrial diameter; LVEDD, left ventricular end-diastolic diameter; LVEF, left ventricular ejection fraction; MRA, mineralocorticoid receptor antagonist; NT-proBNP, N-terminal pro-brain natriuretic peptide; NYHA, New York Heart Association; PVC, premature ventricular contractions; RVD, right ventricular diameter.

### 3.2 Principal Component Analysis and Phenomapping

**Fig. 1.**
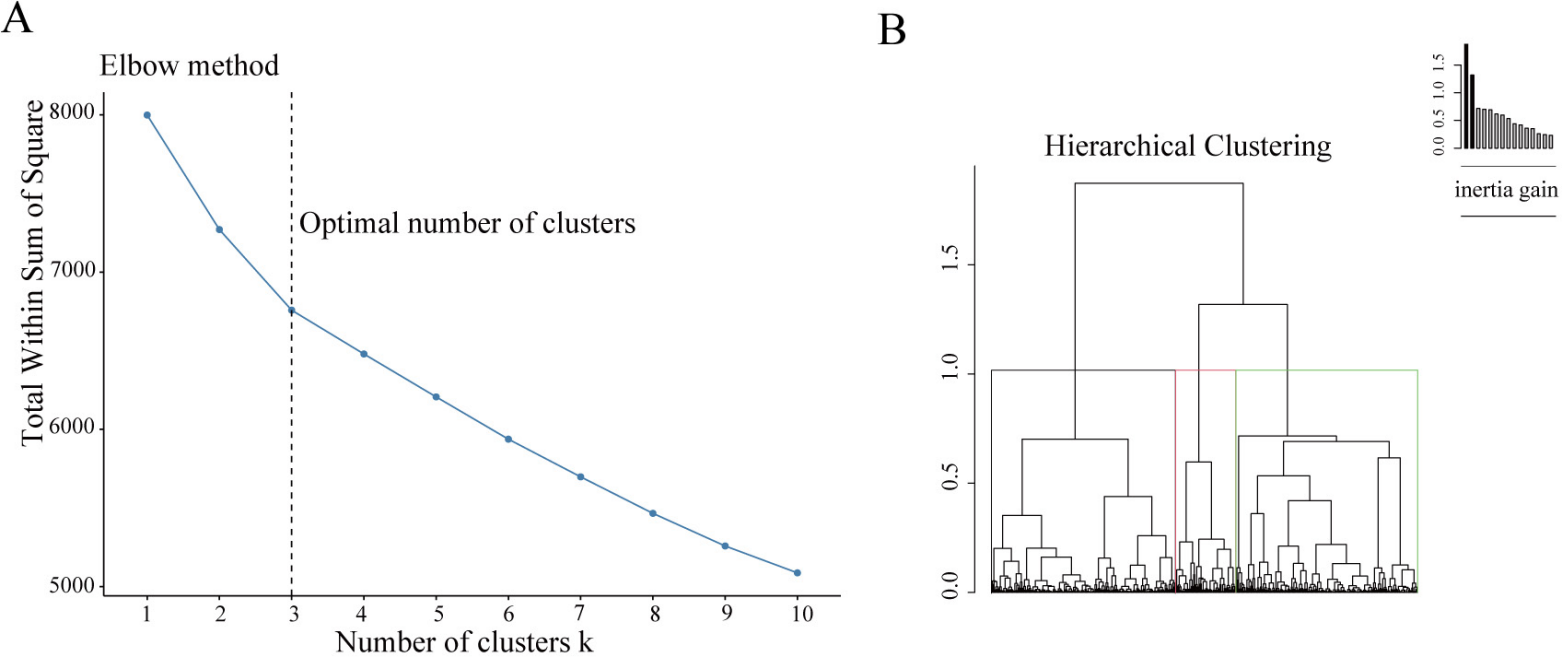
**Building the hierarchical clustering**. (A) The Elbow method to identify the ideal number of clusters. The optimal number of clusters was three, as illustrated. (B) The dendrogram construction.

**Fig. 2.**
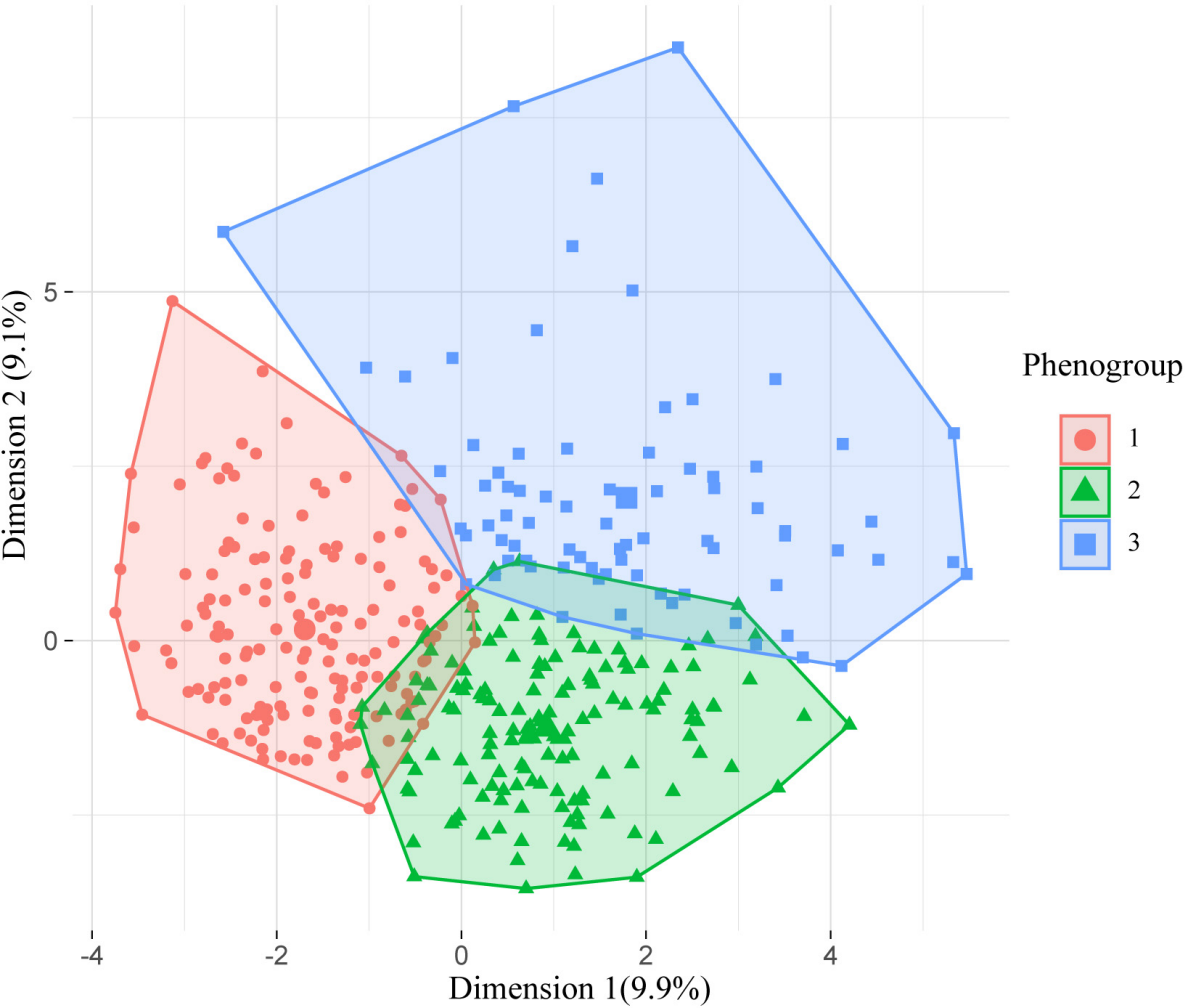
**Visualization of the phenomapping after k-means consolidation**. The X and Y axes represent the first and second extracted principal components, respectively.

The scree plot obtained using FAMD analysis is illustrated in **Supplementary Fig. 1**. The first 12 principal components with eigenvalues >1 and a cumulative explained variance of 60.5% were used for clustering. The first and second principal components accounted for 9.9% and 9.1% of the variation, respectively. The variables that contributed the most to these two dimensions, as illustrated in **Supplementary Fig. 2**, were cardiomyopathy etiology and renal function parameters. Phenomapping was based on the extracted components. The Elbow method presented in Fig. 1 suggests that the best number of clusters was three in hierarchical clustering. Subsequently, the k-means consolidation was performed. As illustrated in Fig. 2, the patients were stratified into three distinct phenogroups. Phenogroups 1, 2, and 3 included 163, 148, and 78 patients, respectively.

### 3.3 Baseline Characteristics among Phenogroups

**Fig. 3.**
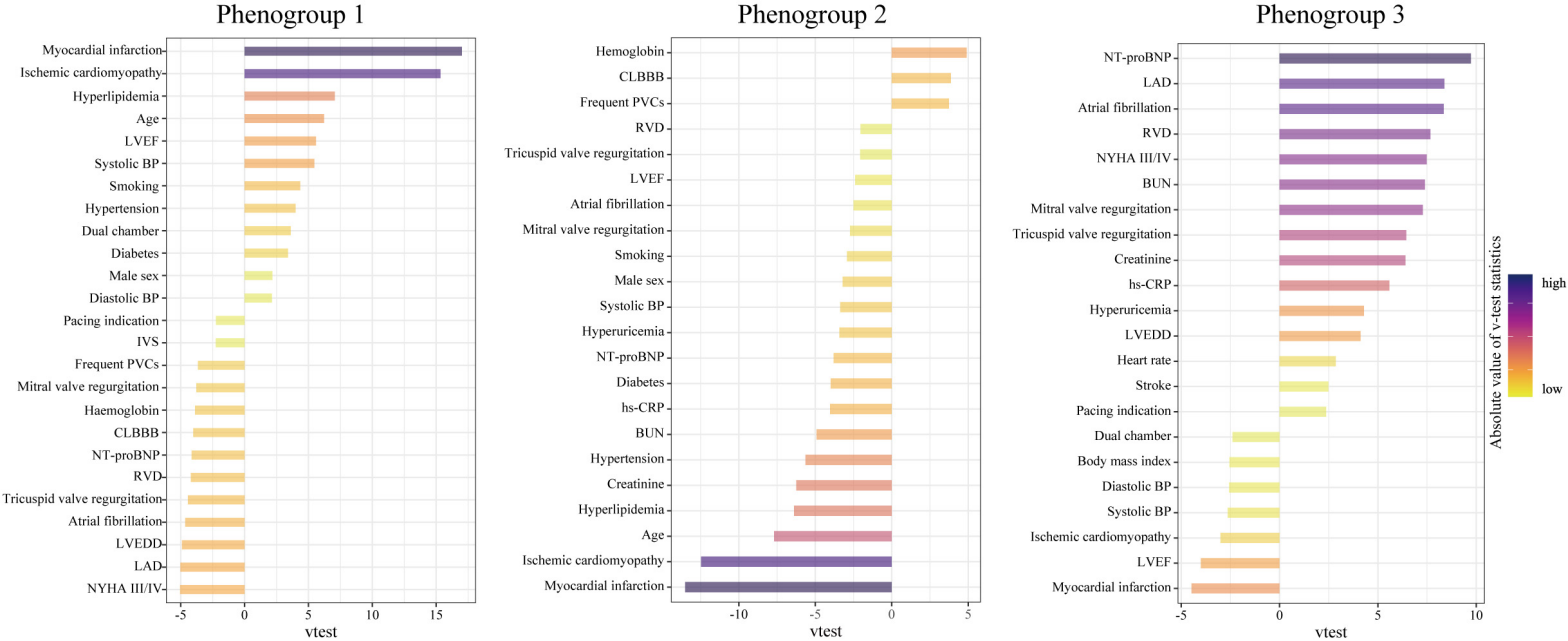
**Characteristics of the three phenogroups by their major representative features**. A higher v-test statistic means a more remarkable representation of a feature compared to the overall population. Positive and negative values represent overrepresentation and underrepresentation, respectively. Abbreviations as in Table 1.

The most representative features of each phenogroup are illustrated in Fig. 3. By adding the group differences presented in Table 1, these three clinical phenogroups were well defined. Phenogroup 1 was the oldest (65.2 ± 10.5 years), and nearly all (95.1%) had ischemic cardiomyopathy. These patients were the most likely to be smokers (64.4%) and had the most favorable cardiac dimensions (left ventricular end-diastolic diameter: 62.0 ± 7.6 mm; left atrial diameter: 42.0 ± 5.5 mm; right ventricular diameter: 21.9 ± 2.9 mm) and function (LVEF: 38.3 ± 7.5%) among the phenogroups. Consistent with these findings, these patients had the highest prevalence of cardiovascular and metabolic comorbidities (diabetes mellitus, hypertension, and hyperlipidemia), the lowest levels of N-terminal pro-brain natriuretic peptide (NT-proBNP) [805.0 (329.0–1787.0) pmol/L], and were the least symptomatic as assessed using the NYHA (NYHA III/IV, 31.3%). Conversely, phenogroup 2 included the youngest patients (54.3 ± 12.4 years) and was predominately composed of non-ischemic cardiomyopathy (86.5%). These patients had intermediate cardiac structure and function; however, the overall best health condition was reflected by the highest hemoglobin, the lowest creatinine and blood urea nitrogen levels, and the lowest burden of comorbidities across phenogroups. Phenogroup 3 exhibited the most severe HF progression, as evidenced by the largest cardiac dimensions, lowest LVEF, and highest rates of atrial fibrillation. In addition, NT-proBNP levels increased approximately threefold compared to the other groups. Moreover, these patients had the highest renal function parameters, indicating the worst cardiac and renal function status among the phenogroups. Based on these features, individuals in phenogroup 3 were more often prescribed diuretics, digoxins, and anticoagulants. However, beta-blocker use was disproportionately low in these patients.

### 3.4 Association of Phenogroups with Clinical Outcomes

**Fig. 4.**
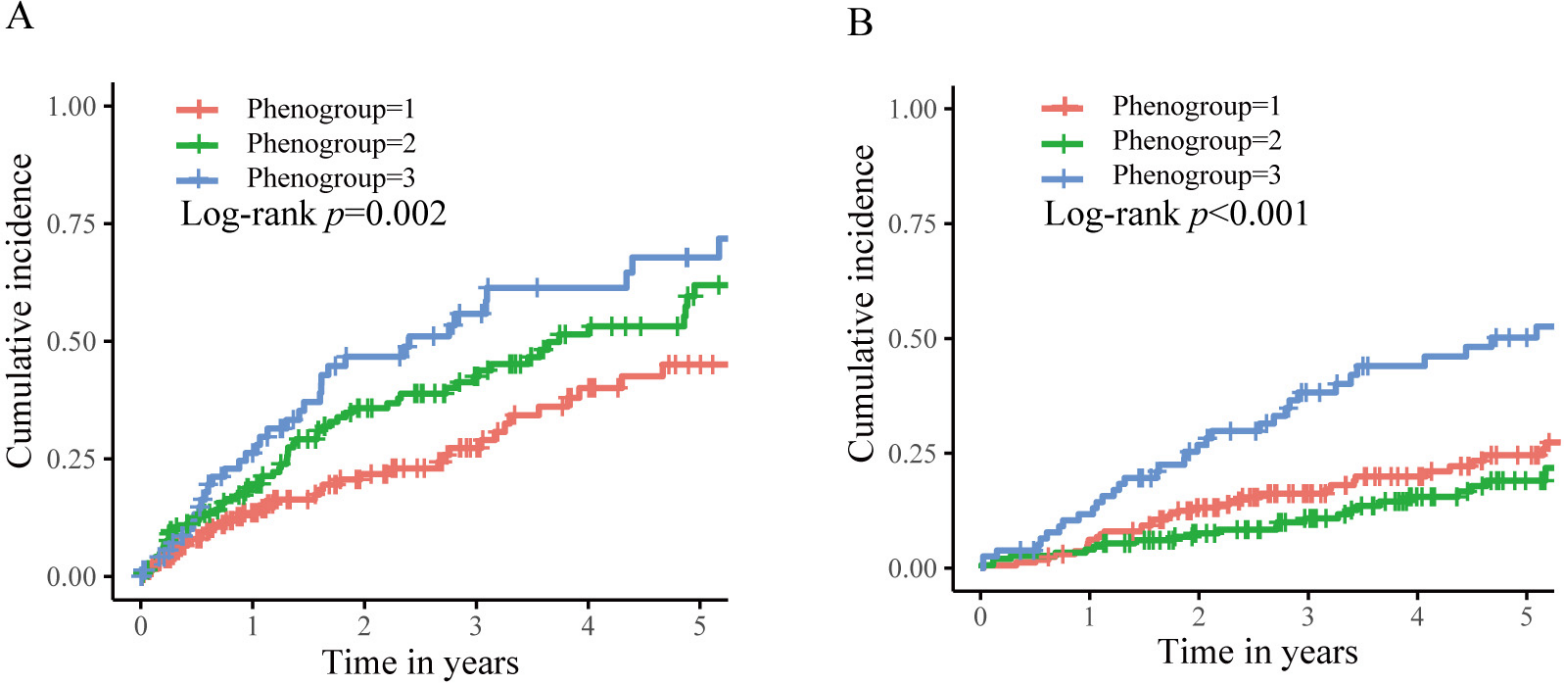
**Kaplan–Meier curves for the clinical outcomes stratified by phenogroups**. (A) The first appropriate shock. (B) The all-cause death.

During a median follow-up of 2.7 years for device interrogation and 5.1 years for survival status, 142 (36.5%) first appropriate shocks and 113 (29.0%) all-cause deaths occurred. As illustrated in Fig. 4, long-term outcomes differed significantly among the phenogroups. Phenogroup 1 had a 5-year cumulative incidence of appropriate shock and all-cause mortality of 45.0% and 24.6%, respectively. Subsequently, they were assigned as the reference group. Table 2 summarizes the unadjusted and adjusted differences between phenogroups. After adjusting for medications in multivariable Cox regression, phenogroups 2 and 3 had a graded increase in the risk of appropriate shock (HR 1.54, 95% CI 1.03–2.28, *p* = 0.033; HR 2.21, 95% CI 1.42–3.43, *p <* 0.001, respectively; *p* for trend <0.001) compared to phenogroup 1. Regarding all-cause death, compared to phenogroup 1, phenogroup 3 was associated with an increased risk (HR 2.25, 95% CI 1.45–3.49, *p <* 0.001), while phenogroup 2 had a comparable risk (HR 0.70, 95% CI 0.44–1.11, *p* = 0.124).

**Table 2.** **Association between phenogroups and clinical outcomes**.

	Phenogroup 1 (n = 163)		Phenogroup 2 (n = 148)			Phenogroup 3 (n = 78)			*p* for trend
	Events	HR⋆	Events	HR (95% CI)	*p*-value	Events	HR (95% CI)	*p*-value	
Unadjusted									
The first appropriate shock	41 (25.2%)	1	63 (42.6%)	1.59 (1.07–2.35)	0.021	38 (48.7%)	2.16 (1.39–3.36)	<0.001	<0.001
All-cause mortality	43 (26.4%)	1	32 (21.6%)	0.68 (0.43–1.09)	0.109	38 (48.7%)	2.24 (1.44–3.47)	<0.001	-
Adjusted									
The first appropriate shock	-	1	-	1.54 (1.03–2.28)	0.033	-	2.21 (1.42–3.43)	<0.001	<0.001
All-cause mortality	-	1	-	0.70 (0.44–1.11)	0.124	-	2.25 (1.45–3.49)	<0.001	-

⋆ Phenogroup 1 was used as the reference. The adjusted analysis included the following medication prescriptions: ACEI/ARB/ARNI, amiodarone, beta-blockers, calcium channel blockers, diuretics, MRA, digoxin, statin, antiplatelet, and anticoagulants. HR, hazard ratio; CI, confidence interval. The other abbreviations are listed in Table 1.

## 4. Discussion

In this follow-up study of secondary ICD recipients with HF, we identified three phenotypically and prognostically distinct phenogroups using principal component analysis and unsupervised phenomapping. Phenogroup 1 comprised the oldest patients with ischemic cardiomyopathy and had the highest diabetes, hypertension, and hyperlipidemia burden. Phenogroup 2 was characterized by the youngest age, non-ischemic cardiomyopathy, and lowest burden of comorbidities. Phenogroup 3 had the worst HF progression. In addition, in phenogroups 1–3, heart structure and function deteriorated. In accordance with these findings, the risk of appropriate shock doubled from phenogroups 1 to 3. Not surprisingly, no VA recurrence-free group was observed. The death risk also doubled in phenogroup 3 compared to that in phenogroup 1. These results offer a novel perspective on predicting the long-term outcomes of different HF subgroups that received secondary ICD implantation and might serve as a triage for these patients.

Prior studies using traditional statistical analysis failed to construct risk assessment tools for secondary ICD recipients [11, 12]. This may be largely explained by the complex pathophysiological interactions underlying VA [13, 18]. Therefore, instead of finding a specific mode between baseline features and prognosis, unsupervised machine learning phenomapping by classifying patients into several clusters based on their similarities may represent a promising alternative [14]. Patients within a cluster, also called a phenogroup, share similar characteristics and prognostic outcomes, whereas patients from different clusters differ [19, 20, 21]. Phenomapping has been utilized extensively in cardiovascular medicine, including the categorization of HF comorbidities [22], classification of patients with HF with preserved ejection fraction [20, 21], dilated cardiomyopathy [17], and identification of cardiac resynchronization therapy responders [19, 23]. To date, there is no gold standard for selecting an optimal algorithm for phenomapping. However, in this study, k-means consolidated hierarchical clustering based on principal components was chosen because of its reliability and effectiveness [15, 16, 17]. Notably, FAMD was used first to extract principal components from mixed data, which also contributed to reducing the redundancy of the raw variables. These factors led to robust clustering, as demonstrated by the unique clinical features and prognoses observed across these phenogroups.

Assessing the risk of VA recurrence and ICD shock is essential to optimize the management of patients with ICD [10, 13]. On the one hand, although life-saving, the ICD shock is related to a higher risk of death, decreased quality of life, and psychiatric disorders, such as anxiety and depression [1, 2]. On the other hand, intensified therapies should not be encouraged indiscriminately, as antiarrhythmic medications have adverse side effects and may increase mortality [24], while VA ablation in ICD recipients has been inconsistent with VA recurrence and mortality [25, 26]. Therefore, it is strongly advised to identify high-risk individuals and take more aggressive actions in these patients, including personalized device programming to reduce appropriate yet unnecessary ICD shocks, innovative drugs (such as sodiumc-glucose cotransporter-2 inhibitors [27]), and catheter ablation to reduce VA recurrence. Aging, male sex [12], VT as a presenting arrhythmia [11, 12, 28], incomplete revascularization [29, 30], and cardiac remodeling [11, 29, 31] are risk factors for VA occurrence in secondary prevention; however, no risk models have been developed. In contrast, we identified three phenogroups with a gradient increase in shock risk parallel with progressive cardiac remodeling and function, which might aid in real-world risk stratification. This finding is also consistent with previous findings that deteriorated left ventricular dimension and function contribute to the increased risk of VA [11, 29, 31, 32].

In addition to identifying the risk of appropriate shock, this phenomapping can also classify the risk of all-cause death. Phenogroup 3 had a higher mortality risk than phenogroup 1, which is consistent with HF worsening. Conversely, phenogroup 2 had a lower risk of death than phenogroup 1, although the difference was not significant. At first glance, this is obscure. However, individuals in phenogroup 2 were much younger (11 years younger) and had a better overall health status, except for heart conditions. Thus, the rate of non-cardiac death might be lower in phenogroup 2 than that of patients in phenogroup 1, which ultimately contributes to a lower risk of all-cause mortality. Notably, ICD is merely designed to terminate the VA. Nevertheless, it can neither reverse pump failure nor prevent non-cardiac deaths. Furthermore, data have revealed that patients with comorbid hypertension [33], renal dysfunction [34], and diabetes [35, 36] derive less or no benefit from ICD implantation. Therefore, it is imperative to minimize non-cardiac deaths among ICD recipients to achieve a mortality benefit. Specifically, for patients in phenogroup 1, optimized control of diabetes, hypertension, hyperlipidemia, and renal function protection should be implemented.

The risk of cardiac death, including pump failure death and SCD, increases as HF worsens [32, 37]. Therefore, it is reasonable to identify individuals with a higher likelihood of SCD than pump failure to maximize ICD implantation benefits [38, 39, 40]. However, defining the extent of HF progression that meets this condition is challenging. Therefore, phenomapping patients with varying degrees of comorbidities and HF is a feasible alternative strategy. For instance, phenogroup 2 had a lower risk of death but a higher risk of appropriate shock than phenogroup 1. In this case, phenogroup 2 may derive greater benefits from ICD therapy. Notably, as our patients exclusively comprised secondary prevention indications, no subgroup of patients could be deemed as having no need for ICD implantation. Except for identifying those most likely or unlikely to benefit from ICD, phenomapping is also paramount in the historical period when subcutaneous ICD (S-ICD) is gaining momentum. The S-ICD is designed to overcome some transvenous ICD-related complications, such as vascular injury, lead failure, and systemic infections; however, it is not free from device-related complications and inappropriate shocks [41]. Moreover, there remains a need for a certain population implanted with S-ICD to upgrade to a transvenous ICD due to the need for anti-bradycardia pacing, anti-tachycardia pacing, or cardiac resynchronization therapy [42]. Phenomapping techniques can identify patients suitable for S-ICD implantation.

Our study has some limitations. It was conducted exclusively in a tertiary hospital; therefore, the conclusions drawn not be directly applicable to external datasets. Therefore, validation of phenomapping is required. Nonetheless, as these phenogroups are well characterized and resemble real-world situations, phenomapping can be inferred to be robust. Second, no standard protocol for device programming was used, and parameter tuning might have occurred during the follow-up. Nevertheless, shock therapies were generally triggered after ATP failed to terminate the VA. Therefore, an appropriate shock could be considered a reliable surrogate for lethal VA. Third, due to the difficulties of adjudication, we did not distinguish between deaths caused by HF progression, SCD, or non-cardiac causes. However, determining the cause of death would undoubtedly provide a more comprehensive perspective for interpreting this phenomapping. Fourth, the patients were undertreated with guideline-directed medical therapy for HF at baseline. Nevertheless, given that phenomapping is solely based on baseline features, except for medications, and computed by similarities between individual patients, outcome differences between phenogroups were unlikely to change significantly even after modifying medications. Finally, only the routine clinical variables were included. Genetic testing, inflammatory markers, computed tomography, and cardiac magnetic resonance parameters were unavailable. This hinders the possibility of diving into a deeper understanding of the phenomapping.

## 5. Conclusions

As a proof-of-concept study, we have demonstrated that unsupervised machine learning phenomapping based on principal components is a promising way to identify subgroups of patients with HF implanted with ICD, among whom clinical characteristics and prognosis substantially differed. This novel approach has the potential to facilitate risk stratification and guide the individualized management of patients with ICD.

## Data Availability

The datasets generated and/or analyzed during the current study are not publicly available due to the regulation of Fuwai Hospital.

## Abbreviations

FAMD, factor analysis of mixed data; HF, heart failure; ICD, implantable cardioverter-defibrillator; LVEF, left ventricular ejection fraction; NT-proBNP, N-terminal pro-brain natriuretic peptide; NYHA, New York Heart Association; SCD, sudden cardiac death; VA, ventricular arrhythmias.

## Supplementary Material

Supplementary material associated with this article can be found, in the online version, at https://doi.org/10.31083/j.rcm2402037.

## References

[1] Priori SG, Blomstrom-Lundqvist C, Mazzanti A, Blom N, Borggrefe M, Camm J (2015). 2015 ESC Guidelines for the management of patients with ventricular arrhythmias and the prevention of sudden cardiac death: The Task Force for the Management of Patients with Ventricular Arrhythmias and the Prevention of Sudden Cardiac Death of the European Society of Cardiology (ESC). Endorsed by: Association for European Paediatric and Congenital Cardiology (AEPC). *European Heart Journal*.

[2] Al-Khatib SM, Stevenson WG, Ackerman MJ, Bryant WJ, Callans DJ, Curtis AB (2018). 2017 AHA/ACC/HRS guideline for management of patients with ventricular arrhythmias and the prevention of sudden cardiac death: Executive summary: A Report of the American College of Cardiology/American Heart Association Task Force on Clinical Practice Guidelines and the Heart Rhythm Society. *Heart Rhythm*.

[3] van Welsenes GH, van Rees JB, Borleffs CJW, Cannegieter SC, Bax JJ, van Erven L (2011). Long-term follow-up of primary and secondary prevention implantable cardioverter defibrillator patients. *Europace*.

[4] Reeder HT, Shen C, Buxton AE, Haneuse SJ, Kramer DB (2019). Joint Shock/Death Risk Prediction Model for Patients Considering Implantable Cardioverter-Defibrillators. *Circulation: Cardiovascular Quality and Outcomes*.

[5] Zabel M, Willems R, Lubinski A, Bauer A, Brugada J, Conen D (2020). Clinical effectiveness of primary prevention implantable cardioverter-defibrillators: results of the EU-CERT-ICD controlled multicentre cohort study. *European Heart Journal*.

[6] Bilchick KC, Wang Y, Cheng A, Curtis JP, Dharmarajan K, Stukenborg GJ (2017). Seattle Heart Failure and Proportional Risk Models Predict Benefit from Implantable Cardioverter-Defibrillators. *Journal of the American College of Cardiology*.

[7] Wu KC, Wongvibulsin S, Tao S, Ashikaga H, Stillabower M, Dickfeld TM (2020). Baseline and Dynamic Risk Predictors of Appropriate Implantable Cardioverter Defibrillator Therapy. *Journal of the American Heart Association*.

[8] Zegard A, Okafor O, de Bono J, Kalla M, Lencioni M, Marshall H (2021). Myocardial Fibrosis as a Predictor of Sudden Death in Patients with Coronary Artery Disease. *Journal of the American College of Cardiology*.

[9] Oscar O, Enrique R, Andres B (2004). Subanalyses of secondary prevention implantable cardioverter-defibrillator trials: antiarrhythmics versus implantable defibrillators (AVID), Canadian Implantable Defibrillator Study (CIDS), and Cardiac Arrest Study Hamburg (CASH). *Current Opinion in Cardiology*.

[10] Borne RT, Katz D, Betz J, Peterson PN, Masoudi FA (2017). Implantable Cardioverter‐Defibrillators for Secondary Prevention of Sudden Cardiac Death: a Review. *Journal of the American Heart Association*.

[11] Borleffs CJ, van Erven L, Schotman M, Boersma E, Kies P, van der Burg AE (2009). Recurrence of ventricular arrhythmias in ischaemic secondary prevention implantable cardioverter defibrillator recipients: long-term follow-up of the Leiden out-of-hospital cardiac arrest study (LOHCAT). *European Heart Journal*.

[12] Schaer B, Kühne M, Reichlin T, Osswald S, Sticherling C (2016). Incidence of and predictors for appropriate implantable cardioverter-defibrillator therapy in patients with a secondary preventive implantable cardioverter-defibrillator indication. *Europace*.

[13] Nielsen JC, Lin YJ, de Oliveira Figueiredo MJ, Sepehri Shamloo A, Alfie A, Boveda S (2020). European Heart Rhythm Association (EHRA)/Heart Rhythm Society (HRS)/Asia Pacific Heart Rhythm Society (APHRS)/Latin American Heart Rhythm Society (LAHRS) expert consensus on risk assessment in cardiac arrhythmias: use the right tool for the right outcome, in the right population. *Europace*.

[14] Feeny AK, Chung MK, Madabhushi A, Attia ZI, Cikes M, Firouznia M (2020). Artificial Intelligence and Machine Learning in Arrhythmias and Cardiac Electrophysiology. *Circulation: Arrhythmia and Electrophysiology*.

[15] Husson F, Josse J, Pages J (2010). Principal component methods-hierarchical clustering-partitional clustering: why would we need to choose for visualizing data. *Applied Mathematics Department*.

[16] Kassambara A (2017). Practical guide to principal component methods in R: PCA, M (CA), FAMD, MFA, HCPC, factoextra. http://www.sthda.com.

[17] Verdonschot JAJ, Merlo M, Dominguez F, Wang P, Henkens MTHM, Adriaens ME (2021). Phenotypic clustering of dilated cardiomyopathy patients highlights important pathophysiological differences. *European Heart Journal*.

[18] Halliday BP, Cleland JGF, Goldberger JJ, Prasad SK (2017). Personalizing Risk Stratification for Sudden Death in Dilated Cardiomyopathy: The Past, Present, and Future. *Circulation*.

[19] Cikes M, Sanchez-Martinez S, Claggett B, Duchateau N, Piella G, Butakoff C (2019). Machine learning-based phenogrouping in heart failure to identify responders to cardiac resynchronization therapy. *European Journal of Heart Failure*.

[20] Segar MW, Patel KV, Ayers C, Basit M, Tang WHW, Willett D (2020). Phenomapping of patients with heart failure with preserved ejection fraction using machine learning‐based unsupervised cluster analysis. *European Journal of Heart Failure*.

[21] Shah SJ, Katz DH, Selvaraj S, Burke MA, Yancy CW, Gheorghiade M (2015). Phenomapping for Novel Classification of Heart Failure with Preserved Ejection Fraction. *Circulation*.

[22] Gulea C, Zakeri R, Quint JK (2021). Model-based comorbidity clusters in patients with heart failure: association with clinical outcomes and healthcare utilization. *BMC Medicine*.

[23] Gallard A, Bidaut A, Hubert A, Sade E, Marechaux S, Sitges M (2021). Characterization of Responder Profiles for Cardiac Resynchronization Therapy through Unsupervised Clustering of Clinical and Strain Data. *Journal of the American Society of Echocardiography*.

[24] AbdelWahab A, Sapp J (2017). Ventricular tachycardia with ICD shocks: when to medicate and when to ablate. *Current Cardiology Reports*.

[25] Prasitlumkum N, Navaravong L, Desai A, Desai D, Cheungpasitporn W, Rattanawong P (2022). Impact of early ventricular tachycardia ablation in patients with an implantable cardioverter-defibrillator: an updated systematic review and meta-analysis of randomized controlled trials. *Heart Rhythm*.

[26] Kheiri B, Barbarawi M, Zayed Y, Hicks M, Osman M, Rashdan L (2019). Antiarrhythmic Drugs or Catheter Ablation in the Management of Ventricular Tachyarrhythmias in Patients with Implantable Cardioverter-Defibrillators: A Systematic Review and Meta-Analysis of Randomized Controlled Trials. *Circulation: Arrhythmia and Electrophysiology*.

[27] Samuel TJ, Lai S, Schar M, Wu KC, Steinberg AM, Wei AC (2022). Myocardial ATP depletion detected noninvasively predicts sudden cardiac death risk in patients with heart failure. *JCI insight*.

[28] Boulé S, Sémichon M, Guédon-Moreau L, Drumez É, Kouakam C, Marquié C (2016). Long-term outcome of implantable cardioverter–defibrillator implantation in secondary prevention of sudden cardiac death. *Archives of Cardiovascular Diseases*.

[29] Nombela-Franco L, Iannaccone M, Anguera I, Amat-Santos IJ, Sanchez-Garcia M, Bautista D (2017). Impact of Chronic Total Coronary Occlusion on Recurrence of Ventricular Arrhythmias in Ischemic Secondary Prevention Implantable Cardioverter-Defibrillator Recipients (VACTO Secondary Study): Insights From Coronary Angiogram and Electrogram Analysis. *JACC: Cardiovascular Interventions*.

[30] van der Lingen ACJ, Becker MAJ, Kemme MJB, Rijnierse MT, Spoormans EM, Timmer SAJ (2021). Reversible Cause of Cardiac Arrest and Secondary Prevention Implantable Cardioverter Defibrillators in Patients with Coronary Artery Disease: Value of Complete Revascularization and LGE‐CMR. *Journal of the American Heart Association*.

[31] Lee W, Chen H, Chen Y, Tsai T, Pan K, Lin Y (2016). Left ventricle remodeling predicts the recurrence of ventricular tachyarrhythmias in implantable cardioverter defibrillator recipients for secondary prevention. *BMC Cardiovascular Disorders*.

[32] Aleong RG, Mulvahill MJ, Halder I, Carlson NE, Singh M, Bloom HL (2015). Left Ventricular Dilatation Increases the Risk of Ventricular Arrhythmias in Patients with Reduced Systolic Function. *Journal of the American Heart Association*.

[33] Huang H, Deng Y, Cheng S, Zhang N, Cai M, Niu H (2022). Comorbid Hypertension Reduces the Risk of Ventricular Arrhythmia in Chronic Heart Failure Patients with Implantable Cardioverter-Defibrillators. *Journal of Clinical Medicine*.

[34] Waks JW, Higgins AY, Mittleman MA, Buxton AE (2015). Influence of Renal Function on Mortality and Ventricular Arrhythmias in Patients Undergoing first Implantable Cardioverter-Defibrillator Generator Replacement. *Journal of Cardiovascular Electrophysiology*.

[35] Junttila MJ, Pelli A, Kenttä TV, Friede T, Willems R, Bergau L (2020). Appropriate shocks and mortality in patients with versus without diabetes with prophylactic implantable cardioverter defibrillators. *Diabetes Care*.

[36] Rørth R, Dewan P, Kristensen SL, Jhund PS, Petrie MC, Køber L (2019). Efficacy of an implantable cardioverter-defibrillator in patients with diabetes and heart failure and reduced ejection fraction. *Clinical Research in Cardiology*.

[37] Mozaffarian D, Anker SD, Anand I, Linker DT, Sullivan MD, Cleland JGF (2007). Prediction of Mode of Death in Heart Failure: the Seattle Heart Failure Model. *Circulation*.

[38] Levy WC, Lee KL, Hellkamp AS, Poole JE, Mozaffarian D, Linker DT (2009). Maximizing Survival Benefit with Primary Prevention Implantable Cardioverter-Defibrillator Therapy in a Heart Failure Population. *Circulation*.

[39] Shadman R, Poole JE, Dardas TF, Mozaffarian D, Cleland JGF, Swedberg K (2015). A novel method to predict the proportional risk of sudden cardiac death in heart failure: Derivation of the Seattle Proportional Risk Model. *Heart Rhythm*.

[40] Lee DS, Hardy J, Yee R, Healey JS, Birnie D, Simpson CS (2015). Clinical Risk Stratification for Primary Prevention Implantable Cardioverter Defibrillators. *Circulation: Heart Failure*.

[41] Mitacchione G, Schiavone M, Gasperetti A, Viecca M, Curnis A, Forleo GB (2020). Neglected lead tip erosion: An unusual case of S-ICD inappropriate shock. *Journal of Cardiovascular Electrophysiology*.

[42] Gasperetti A, Schiavone M, Vogler J, Laredo M, Fastenrath F, Palmisano P (2022). The need for a subsequent transvenous system in patients implanted with subcutaneous implantable cardioverter-defibrillator. *Heart Rhythm*.

