# Supplementary material for: Machine Learning-Based Phenomapping in Patients with Heart Failure and Secondary Prevention Implantable Cardioverter-Defibrillator Implantation: A Proof-of-Concept Study
Source: Rev Cardiovasc Med. 2023 Feb 2;24(2):37. doi: 10.31083/j.rcm2402037 (PMC11273156; doi:10.31083/j.rcm2402037)
Supplement: Supplementary file 1 [file 2153-8174-24-2-037-s1.zip › 2153-8174-24-2-037-s1.docx]

**Machine learning-based** **phenomapping in heart failure patients with secondary prevention** **implantable cardioverter-defibrillator implantation**

**Supplementary material**

**Authors:** Yu Deng, MD; Sijing Cheng, MD; Hao Huang, MD; Xi Liu, MD; Yu Yu, MD; Min Gu, MD; Chi Cai, MD; Xuhua Chen, MD; Hongxia Niu, MD; Wei Hua, MD, Ph.D., FHRS

**Affiliation:** The Cardiac Arrhythmia Center, State Key Laboratory of Cardiovascular Disease, Fuwai Hospital, National Center for Cardiovascular Diseases, Chinese Academy of Medical Sciences and Peking Union Medical College, No.167 North Lishi Road, Beijing 100037, China

**Correspondence:**

Wei Hua, Ph.D.**;** [drhuaweifw@sina.com](mailto:drhuaweifw@sina.com)

**Supplementary Table 1.** Distribution and proportion of missing variables.

**Supplementary Fig. 1**. The scree plot by the factor analysis of mixed data.

**Supplementary Fig. 2.** Contribution of variables in the first two dimensions.

**Supplementary Table 1.** Distribution and proportion of missing variables.

| Variables | Number of missing values | Percentage (%) |
| --- | --- | --- |
| RVD | 23 | 3.87 |
| hs-CRP | 14 | 2.36 |
| NT-proBNP | 17 | 2.86 |
| IVS | 8 | 1.35 |
| Body mass index | 7 | 1.18 |
| LAD | 2 | 0.34 |
| Systolic BP | 1 | 0.17 |
| Diastolic BP | 1 | 0.17 |
| Heart rate | 1 | 0.17 |

BP, blood pressure; hs-CRP, high-sensitivity C-reactive protein; IVS, interventricular septum thickness; LAD, left atrial diameter; NT-proBNP, N-terminal pro-brain natriuretic peptide; RVD, right ventricular diameter.

**Supplementary Fig. 1**. The scree plot by the factor analysis of mixed data. (A) Eigenvalue, (B) The percentage of explained variance.

**
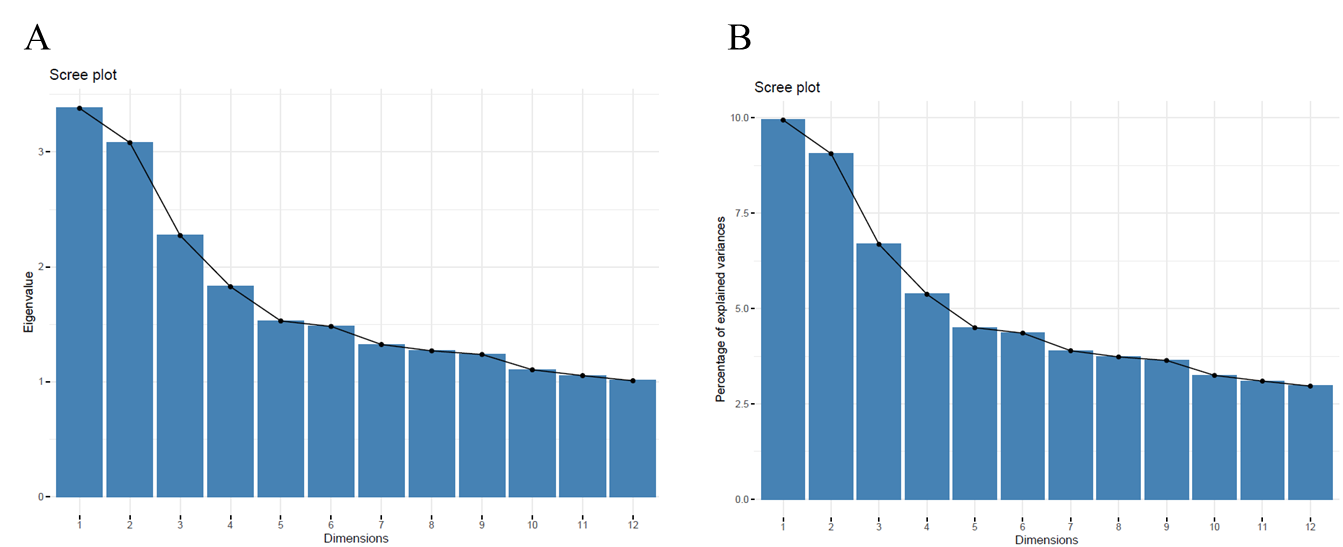
**

**Supplementary Fig. 2.** Contribution of variables in the first two dimensions.


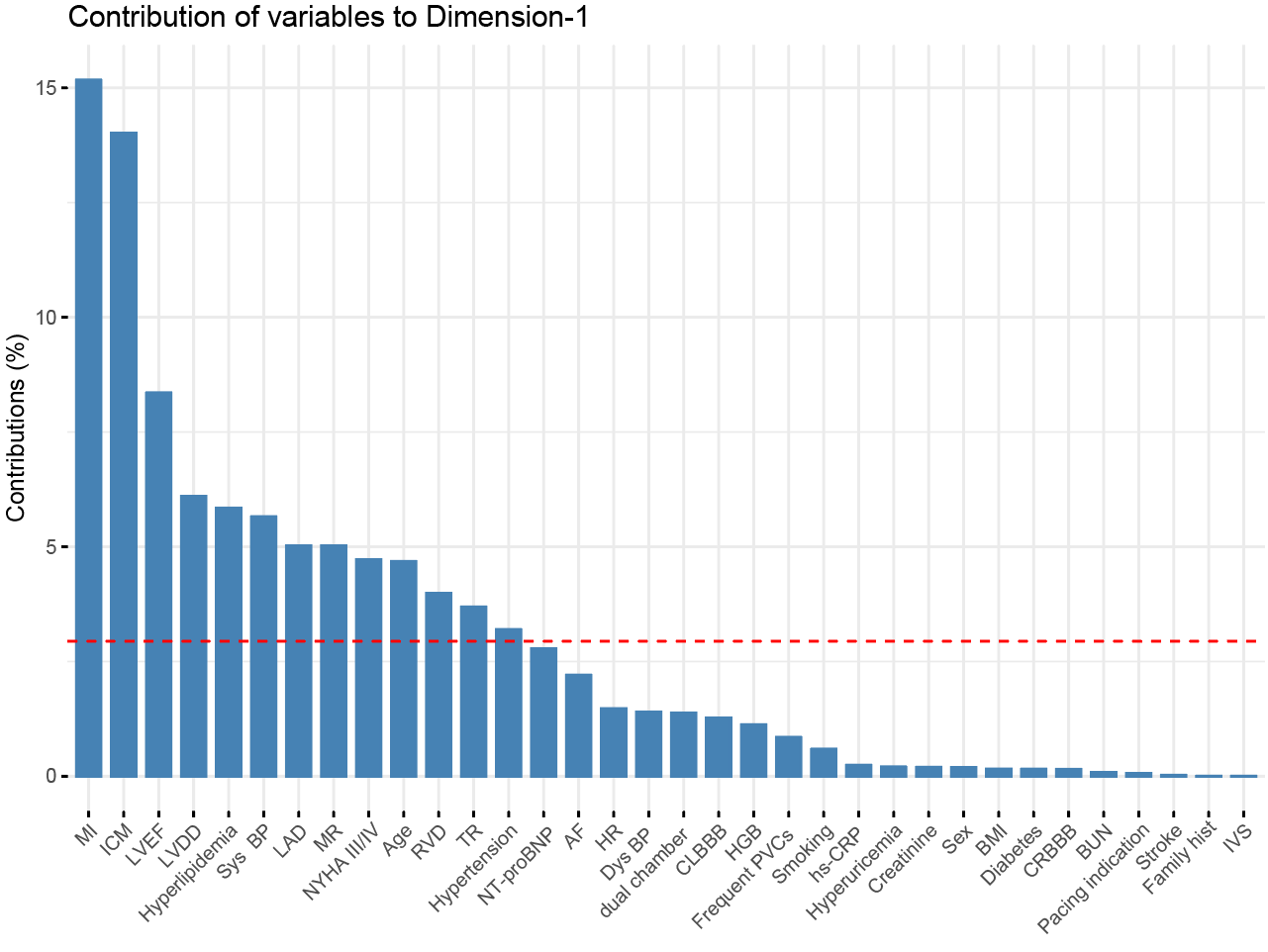


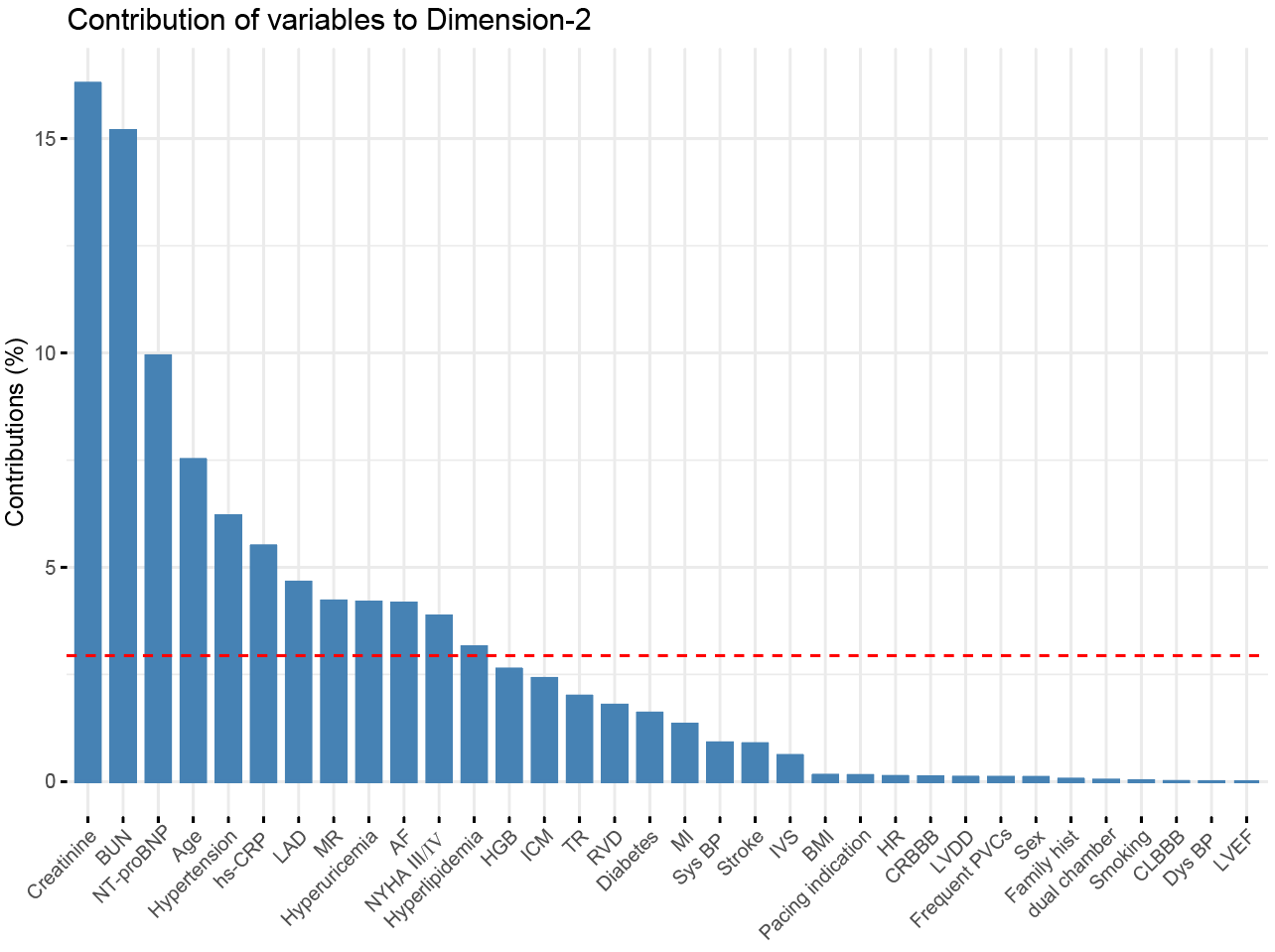


Abbreviations:

AF, atrial fibrillation; BMI, body mass index; BP, blood pressure; BUN, blood urea nitrogen; CLBBB, complete left bundle branch block; CRBBB, complete right bundle branch block; HBG, hemoglobin; HR, heart rate; hs-CRP, high-sensitivity C-reactive protein; IVS, interventricular septum thickness; LAD, left atrial diameter; LVEDD, left ventricular end-diastolic diameter; LVEF, left ventricular ejection fraction; MI, myocardial infarction; MR, mitral valve regurgitation; NT-proBNP, N-terminal pro-brain natriuretic peptide; NYHA, New York Heart Association; PVC, premature ventricular contractions; RVD, right ventricular diameter; TR, tricuspid valve regurgitation.
